# Perimetric and retinal nerve fiber layer findings in patients with Parkinson’s disease

**DOI:** 10.1186/1471-2415-12-54

**Published:** 2012-10-02

**Authors:** Evangelia E Tsironi, Anna Dastiridou, Andreas Katsanos, Efthymios Dardiotis, Stella Veliki, Gianna Patramani, Fani Zacharaki, Stella Ralli, Georgios M Hadjigeorgiou

**Affiliations:** 1 Department of Ophthalmology, University Hospital of Larissa, Faculty of Medicine, University of Thessaly, Mezourlo 41222 Larissa, Greece; 2 Department of Neurology, University Hospital of Larissa, Faculty of Medicine, University of Thessaly, Larissa, Greece

**Keywords:** Visual loss, Visual fields, Parkinson's disease, Retina, Visual processing

## Abstract

**Background:**

Visual dysfunction is common in Parkinson’s disease (PD). It remains, however, unknown whether it is related to structural alterations of the retina. The aim of this study is to compare visual field (VF) findings and circumpapillary retinal nerve fiber layer (RNFL) thickness in a series of PD patients and normal controls, in order to assess possible retinal anatomical changes and/or functional damage associated with PD.

**Methods:**

PD patients and controls were recruited and underwent VF testing with static automated perimetry and RNFL examination with optical coherence tomography (OCT). Cognitive performance using Mini Mental State Examination (MMSE), PD staging using modified Hoehn and Yahr (H-Y) scale and duration of the disease was recorded in PD patients.

**Results:**

One randomly selected eye from each of 24 patients and 24 age-matched controls was included. OCT RNFL thickness analysis revealed no difference in the inferior, superior, nasal or temporal sectors between the groups. The average peripapillary RNFL was also similar in the two groups. However, perimetric indices of generalized sensitivity loss (mean deviation) and localized scotomas (pattern standard deviation) were worse in patients with PD compared to controls (p < 0.01). 73% of eyes of PD patients had glaucomatous-like asymmetrical hemifield defects with abnormal Glaucoma Hemifield Test and various combinations of arcuate defects (n = 12), nasal steps (n = 11) and paracentral scotomas (n = 16). Bilateral defects were found in 14 patients (58%). No correlation was found between VF indices and MMSE or H-Y scores.

**Conclusion:**

PD patients may demonstrate glaucomatous-like perimetric defects even in the absence of decreased RNFL thickness.

## Background

Parkinson’s disease (PD) is a neurodegenerative disorder characterized by selective loss of dopaminergic neurons in the nigrostriatal pathway. PD patients experience progressive motor impairment, often accompanied by a variety of non-motor symptoms [1]. Visual dysfunction is common in PD and manifests as visual acuity loss, reduced colour discrimination and deficiencies in visual contrast sensitivity and motion perception [2-6]. These visual deficits may stem from dopaminergic denervation of amacrine retinal cells [6,7]. Dopamine is essential for light adaptation, by facilitating visual signal transmission in rod and cone circuits, and exerts multiple trophic effects in retinal cells [7]. Studies in autopsy cases of PD revealed loss of the dopaminergic innervation around the fovea and decreased retinal dopamine concentration despite the preservation of retinal dopaminergic neurons [8,9]. It remains, however, unknown whether these changes could result in structural alterations of the retina or only in functional deficits.

In a search of a potential biomarker of PD progression or response to neuroprotective agents, a number of studies [10-12] using optical coherence tomography (OCT), a non-invasive imaging modality [13], have provided evidence of peripapillary retinal nerve fiber layer (RNFL) thinning in PD patients. However, recent studies did not confirm the presence of structural alterations of the retina around the optic nerve head in PD patients [14-16]. On the contrary, visual function tests have highlighted differences between PD patients and controls [6]. A previous study has shown characteristic visual field (VF) defects in PD patients [17].

In the present study, in order to investigate both retinal anatomical changes and functional damage in PD, we compared RNFL thickness measured with OCT and VFs performed with static automated perimetry in a series of PD patients and normal controls.

## Methods

### Patients

This cross-sectional case–control study was conducted at the University Eye Clinic of Larissa, Greece, between June 2009 and June 2010. Patients diagnosed with PD attending the University Neurology Clinic of Larissa, Greece, were referred for ophthalmic evaluation in the University Ophthalmology Department. Patients were recruited in a consecutive-if-eligible fashion. All patients in our sample underwent a neurologic examination. Eligible patients met the criteria for PD [18] including substantial and sustained response to L-dopa, and were under stable and optimal treatment with L-dopa. In addition, all patients were receiving treatment and were in “ON” period during the ophthalmic evaluation. The patients’ duration of PD, general medical history and treatment modalities were recorded. PD staging was assessed with the modified Hoehn and Yahn (H-Y) scale and severity of cognitive impairment was evaluated using the Mini Mental State Examination (MMSE) test. Age- and gender-matched individuals attending the outpatient eye clinic for mild cataracts or refraction problems were recruited as controls.

Inclusion criteria for PD patients and controls were: visual acuity equal to or better than 6/10, spherical equivalent refractive error between −5 and +3 diopters, at least two intraocular pressure (IOP) readings lower than 21 mmHg at two different time points, and ability to follow the study protocol. In an effort to avoid confounding conditions, exclusion criteria were: presence of diabetic retinopathy, presence of other neurodegenerative disorders, positive family history of glaucoma, narrow anterior chamber angles at gonioscopy, cup-to-disc ratio greater than 0.6, glaucomatous appearance of the optic discs (defined as fellow eye asymmetry in cup-to-disc ratio > 0.2, neuroretinal rim thinning, disc hemorrhages or retinal nerve fiber layer defects), signs of ocular disease other than visually non-significant cataract, and previous ocular surgery other than uneventful phacoemulsification.

### Procedures

Both PD patients and controls underwent a thorough ophthalmic evaluation, including assessment of best-corrected visual acuity, VF testing, Goldmann applanation tonometry, slit lamp examination, gonioscopy and dilated fundoscopy with a 90 diopters lens. Standard automated perimetry was performed in all patients with the Humphrey 24–2 SITA-Standard algorithm on the day of examination and was repeated after a period of up to two weeks. RNFL OCT scans were obtained in all participants after pupil dilation in the first visit, using the Stratus OCT (Carl Zeiss Meditec, Dublin, CA, USA) and analyzed with the fast RNFL algorithm. Only participants with high quality OCT scans (signal strength ≥7) and no artifacts were included.

To be eligible for analysis, participants had to have a reliable second VF (false positive and false negative responses lower than 33% and fixation losses lower than 20%). Mean deviation (MD) and pattern standard deviation (PSD) were used as perimetric indices of generalized sensitivity loss and localized scotomas respectively. Only data from the second VF entered the statistical analysis. Based on reports about scotomas similar to glaucomatous visual field losses in PD patients [17], in addition to the Humphrey-provided MD and PSD indices, we calculated sensitivity scores over the superior and inferior hemifields. The VF printout was used for the calculation of mean sensitivity scores in the superior (Mean Sensitivity Superior-MSS) and inferior hemifield (Mean Sensitivity Inferior-MSI) from the individual test-point sensitivity values in the corresponding hemifield. The Glaucoma Hemifield Test (GHT) results, as an index of asymmetry between sensitivity losses in the superior and inferior hemifield of each eye were also analyzed. A scotoma was defined as a cluster of at least three abnormal points (including two or more points depressed by a p-value less than 0.5%) on the pattern deviation probability map of the Humphrey 24–2 SITA-Standard programme. Points with decreased sensitivity immediately adjacent to the blind spot were not considered parts of scotomas. Test points at the periphery (rim points) could be considered as parts of scotomas if at least two of the points were non-peripheral (nonrim) [19].

The study protocol adhered to the tenets of the Helsinki Declaration and was approved by the Institutional Board of the University Hospital of Larissa, Greece. All participants gave informed consent before entering the study.

### Statistical analysis

Both eyes from each patient were examined. With the exception of the VF asymmetry analysis in PD patients, one randomly selected eye from each participant was used for all other comparisons [20]. Data are presented as mean ± standard deviation (SD). Normality of distribution was assessed with the Kolmogorov-Smirnov test. The independent samples t-test was used in comparisons of normally distributed data. Mann–Whitney testing was used for non-parametric comparisons. The chi-square test was used to compare differences between groups in categorical variables. Comparisons between fellow eyes or between superior and inferior hemifields of the same eye were performed with the paired t-test where appropriate, or otherwise with the Wilcoxon signed ranks test. Correlation analysis was performed with the Spearman test. A p-value below 0.05 was considered significant. The SPSS software package was used for all analyses (version 16; SPSS, Inc., Chicago, IL, USA).

## Results

A sample of 33 PD patients was initially recruited, with nine patients being excluded due to unreliable VFs (n = 7) or inability to acquire an acceptable OCT scan (n = 2). Thus, data from 24 PD patients and 24 controls were analyzed. Mean (SD) age was 66.6 (10.2) years in PD patients and 64.3 (7.3) years in the control group (p = 0.374). Mean disease duration, H-Y staging and MMSE score in the PD group were 5.3 (3.5) years, 1.9 (0.6) and 28.3 (2.4), respectively. Mean IOP was 15.3 (2.2) mmHg in the PD and 14.5 (2.6) mmHg in the control group (p = 0.241). Patients’ characteristics are displayed in Table 1 and Additional file 1.

**Table 1:** Characteristics of the study groups. Results presented as mean (standard deviation)

	**PD group**	**Control group**	**p-value**
Age (years)	66.6 (10.2)	64.3 (7.3)	0.374^1^
Sex (men/women)	14/10	11/13	0.386^2^
Right/ Left eye	14/10	13/11	0.771^2^
Decimal Visual Acuity	0.86 (0.12)	0.83 (0.12)	0.363^1^
Intraocular Pressure (mmHg)	15.3 (2.2)	14.5 (2.6)	0.241^1^

PD: Parkinson disease, ^1^: independent samples t-test, ^2^: chi-square test.

Analysis of the OCT parameters revealed no difference between the groups in mean RNFL thickness in the inferior, superior, nasal and temporal sectors of the retina (Table 2).

**Table 2:** Optical coherence tomography and visual field results [mean (standard deviation)] for the study and control groups

		**PD group**	**Control group**	**p-value**
OCT parameters	Superior average (μm)	116.58 (17.09)	122.05 (18.33)	0.312^1^
	Inferior average (μm)	128.58 (16.12)	127.05 (18.55)	0.771^1^
	Nasal average (μm)	72.38 (18.06)	70.35 (8.90)	0.650^1^
	Temporal average (μm)	68.58 (13.91)	67.10 (12.49)	0.714^1^
	Average thickness (μm)	96.42 (11.13)	96.34 (11.46)	0.982^1^
Visual field parameters	Mean Deviation (dB)	−5.71 (4.59)	−0.92 (1.99)	<0.001^1^
	Pattern Standard Deviation (dB)	4.39 (2.59)	2.26 (0.70)	0.013^2^
	Mean Sensitivity Inferior (dB)	23.95 (4.30)	29.64 (5.81)	<0.001^2^
	Mean Sensitivity Superior (dB)	22.17 (5.84)	29.36 (5.74)	<0.001^2^

OCT: Optical coherence tomography.

PD: Parkinson’s disease.

^1^: Independent samples t-test.

^2^: Mann Whitney test.

Reliable VFs were obtained from 48 eyes of 24 PD patients. MD and PSD scores were worse in PD patients compared to the control group (p < 0.05, Table 2). Likewise, MSI and MSS were found to be lower in PD patients, compared to controls (p < 0.01, Table 2). In the PD group, mean MSS was 22.17 (5.84) dB and mean MSI was 23.95 (4.30) dB. MSI was higher compared to MSS (p = 0.014, Wilcoxon signed ranks test).

No correlation was found between MD and MMSE scores (Spearman *ρ* = 0.313, p = 0.137) or H-Y scale scores (*ρ* = −0.229, p = 0.281).

**Figure 1:**
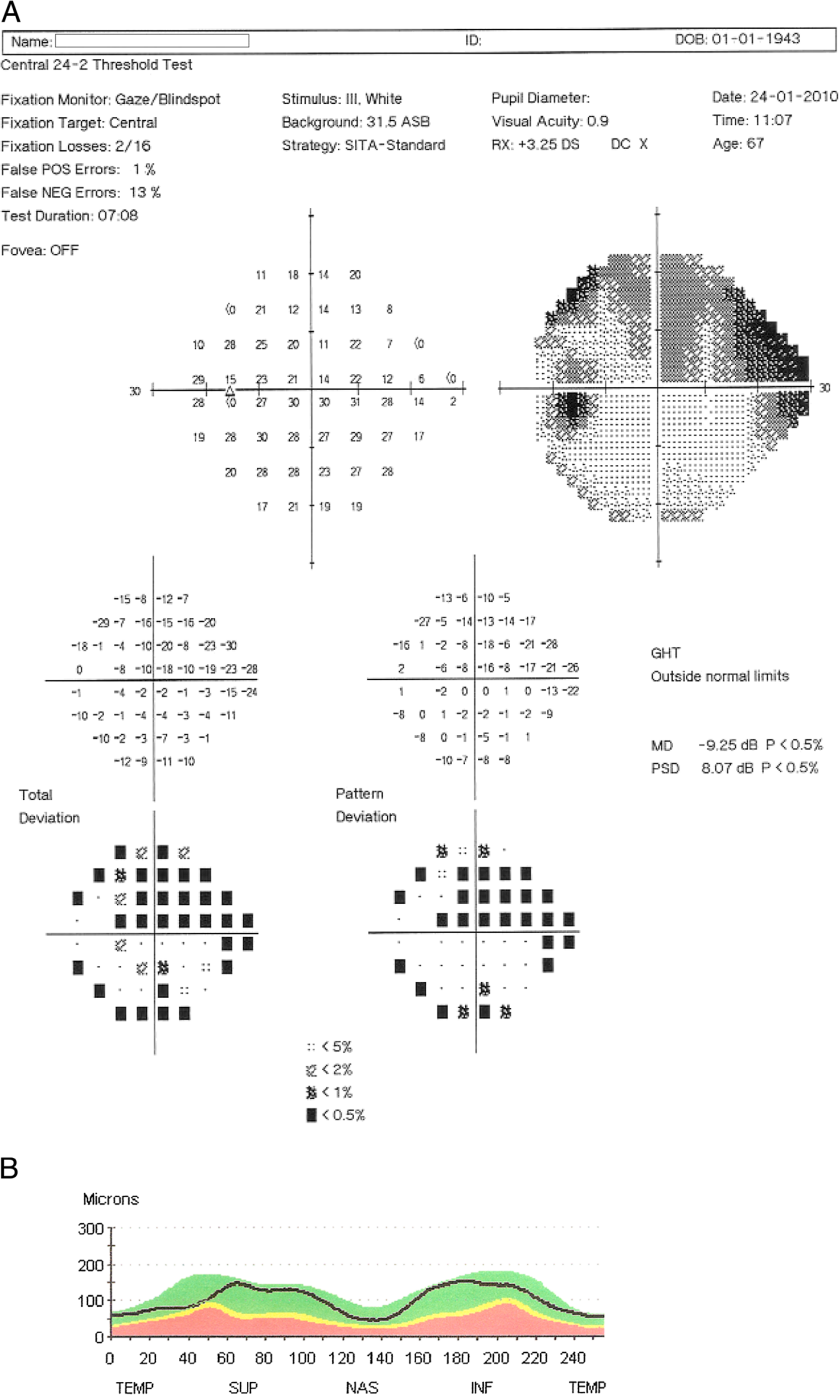
**A and B: Humphrey visual field (A) and peripapillary retinal nerve fiber layer (RNFL) thickness profile (B) of the left eye of a 67 years-old man with Parkinson’s disease. **The visual field print-out shows decreased mean deviation (MD), pathological pattern standard deviation (PSD), glaucoma hemifield test (GHT) outside normal limits, and deep superior arcuate scotoma. The optical coherence tomography printout shows that the RNFL thickness is within normal limits. The marginal decrease of RNFL thickness at the superior-temporal area does not correspond to the visual field scotomas.

In the group of PD patients, the GHT was within normal limits in 8 (16.7%) eyes, borderline in 5 (10.4%) eyes and outside normal limits in 35 (72.9%) eyes. In the group of patients whose GHT was outside normal limits, arcuate defects were evident in 12 eyes, nasal steps in 11 eyes, and paracentral scotomas in 16 eyes (Figure 1). Bilateral defects were found in 14 patients. No VF defects were detected in controls.

Additional analysis was performed to test for asymmetry in VF indices of fellow eyes. Right and left eyes were not found to differ for MD and PSD in PD patients (paired t-test, p = 0.518 and Wilcoxon signed ranks test, p = 0.440, respectively).

## Discussion

The present study did not provide evidence of RNFL thinning in patients with PD. However, VF defects were more frequent in PD patients compared to controls.

A number of previous studies [10-12], although not all [14-16], provided evidence of reduced RNFL thickness in PD patients. Inzelberg et al. [11] reported a reduction in the infero temporal RNFL thickness, which was topographically matched to the VF defects, in a subset of five patients with reliable VFs. A reduction in average RNFL thickness, macular thickness and volume was also reported in another study in PD patients [11]. Decreased RNFL thickness has also been suggested in PD patients examined with scanning laser ophthalmoscopy [21]. Moschos et al. [12] reported reduced temporal and inferior RNFL thickness in PD patients compared to controls. However, in agreement with our results, mean RNFL thickness did not differ between the groups. In addition, OCT findings with spectral domain OCT (SD-OCT) suggest a decreased thickness of the paramacular inner retina, including the nerve fiber layer, the ganglion cell layer and the inner plexiform layer in PD patients, while outer retinal layer thickness was not found to differ from controls [22]. Increased inner nuclear layer thickness has been identified in a more recent study [16].

The finding of RNFL thinning in PD patients has been attributed to the loss of trophic effects induced by dopamine depletion in the retina [7]. Although autopsy studies have documented decreased retinal dopamine concentration in PD patients [9], degeneration of retinal dopaminergic neurons and changes in cell density have not been shown [8]. It is not obvious how dopamine depletion could mediate RNFL thinning in PD. A pilot study using the recently introduced SD-OCT imaging platform did not detect differences in the RNFL thickness of PD patients and controls [14]. Moreover, Archibald et al. failed to detect any RNFL thinning in PD patients, but showed more frequent functional visual defects [15]. In another study in PD patients, no evidence of RNFL thinning with the use of SD- OCT was found [16]. Similarly, in our present study, no significant difference was detected in RNFL thickness in PD patients and controls. Discrepancies between studies may be attributed to differences in study populations, small sample size in some reports, different stages of disease and differences in imaging technologies. In our study, the widely available time-domain OCT platform (Stratus OCT) was used. This device is based on low-coherence interferometry to produce high-resolution, two-dimensional images of the optic nerve head and retina [23]. More recently, SD-OCT, with a scanning speed up to 200 times higher than time-domain OCT and a higher axial resolution was introduced in clinical practice [24-26]. Consequently, it could be argued that SD-OCT would provide more valid data and a greater discriminating ability than time-domain OCT. However, despite the theoretical advantages of the superior reproducibility, resolution and higher scanning speed, recent studies have failed to demonstrate an unequivocal superiority of the commercially available SD-OCT devices over time-domain OCT in the assessment of optic nerve diseases [27-29]. Therefore, the use of the widely available Stratus OCT that employs time-domain technology instead of newer SD-OCT devices may not necessarily limit the validity of our results.

Compared to structural tests, visual function tests may be more relevant in detecting differences between PD patients and controls. Yenice et al. [17] studied VFs in 14 patients with PD, and found decreased MD and PSD scores compared to the control group. In that study, six eyes had nasal steps and six eyes had arcuate defects. A pattern that resembles nerve fiber bundle defects and glaucomatous-like VF loss was also observed in PD patients in our study: 73% of 48 eyes had a GHT outside normal limits, compared to 50% of 28 eyes in the study by Yenice et al. It should be noted that the GHT is an algorithm that was specifically developed for glaucoma. However, we have chosen to analyze GHT results due to the resemblance of typical glaucomatous VF defects with the scotomas found in our non-glaucoma cohort of PD patients. In line with the report by Yenice et al. [17], arcuate defects, nasal steps and paracentral scotomas were also found in our patients. Bilateral VF damage was identified in 14 of our patients.

An increased occurrence of probable glaucoma was reported in a retrospective chart review of 38 patients with PD [30]. To ensure that our participants did not have glaucoma, that could confound our results, we excluded PD patients and controls who had positive family history of glaucoma, narrow anterior chamber angles on gonioscopy or optic disc findings suspicious for glaucoma. Therefore, despite the similarities of glaucomatous scotomas with the scotomas detected in our study, it is quite unlikely that the perimetric findings observed in our patients can be attributed to glaucomatous neuropathy. In glaucoma, there is typically a matching of structural and functional damage. Characteristic VF defects in glaucoma patients can usually be matched to topographically corresponding damage of the optic disc and/or RNFL. The glaucomatous-like visual field defects observed in our series of non-glaucoma PD patients, however, could not be matched to corresponding RNFL thinning (Figure 1). Instead, it is reasonable to assume that the functional deficit observed in this cohort of patients can be explained by intra-retinal, subcortical and cortical neuronal disorganization or injury related to PD.

Animal and human studies have suggested the involvement of the visual pathway in the disease process of PD [31,32]. It was shown that higher cortical visual processes, as well as the retina may be affected [6]. Reduced responses have been reported by means of pattern electroretinograms (pERG) reflecting ganglion cell dysfunction [33,34], as well as flash ERG, indicating outer retina impairment [34]. Multifocal ERG testing, which is believed to mainly reflect bipolar cell activity also revealed differences in PD patients and controls [12]. pERG studies also highlighted changes in retinal ganglion cell function in PD [34-36]. Increased latencies in visual evoked potentials (VEP) were also found, corresponding to a delayed response to visual stimuli due to retinal or post-retinal processing dysfunction [34,37]. This delayed response could at least in part originate from retinal damage, given that dopamine neurons are rare in the visual system at sites other than the retina [5].

Indeed, the pathophysiological basis of the functional visual system impairment in PD has not been fully elucidated. A decrease in dopamine concentration has been found in the retina of patients suffering from PD [9]. Although the role of dopamine in the retinal neural circuitry is not fully understood, there is evidence that dopaminergic deficiency may directly or indirectly affect amacrine, horizontal and retinal ganglion cells and modify the receptive field output of the retina [6,31]. These changes in the coupling between the different cellular systems that form the retinal network could, at least in part, explain the VF defects observed in the present study.

L-dopa therapy in PD patients may also have an effect on visual parameters. Actually, administration of L-dopa in PD patients was found to transiently reverse contrast sensitivity abnormalities, pERG alterations and VEP latencies [6,37]. On the contrary, in our sample, VF defects were identified despite our patients being under optimal L-Dopa treatment and in “ON” period. This suggests that apart from dopamine deficiency, there may also be additional mechanisms accounting for retinal dysfunction in patients with PD.

It should be noted that fixation problems and motor system symptoms can also make VF testing a demanding task for this group of patients. Moreover, it has been shown that reaction times and saccadic eye movements may be affected in PD [38], leading to poor VF performance. However, all patients analyzed in the present study had reliable VFs. Additionally, the 24–2 SITA-Standard algorithm that was used offers the advantage of a relatively short testing time compared to other full-threshold strategies [39]. Another relevant feature of this algorithm is that it continuously monitors the patient’s response rate and adjusts the stimulus presentation rate accordingly [40]. Therefore, motor dysfunction *per se* cannot fully explain the worse perimetric performance of our patients compared to age-matched controls. In addition, visuo-spatial deficits and dysfunction of the visual information processing from the retina to the visual cortex was also reported in PD patients [6] and could contribute to VF defects. However, in the present study, PD patients had a mean MMSE score that corresponds to a normal cognitive function, while 6 patients had a score under the recently suggested cut-off value [41]. Analysis of the data did not show any correlation between the MMSE score and VF indices. This finding may suggest that VF damage in PD patients can be attributed, at least to a certain extent, to retinal dysfunction.

Regardless of the underlying pathophysiological mechanism, our finding that PD patients can have significant visual field defects is clinically relevant. Clinicians need to be aware of the association between this neurodegenerative disorder and visual field deficits. In fact, the attribution of “true” glaucomatous VF defects in PD patients with definite glaucoma can be clinically challenging. In such cases, as previously discussed, careful attention to the matching patterns of structural and functional damage would be critical for the assessment of possible glaucomatous damage. Even more, as judged by the depth and extent of the scotomas, an appreciable functional deficit could be anticipated for at least some of these patients. The impact of these VF defects on the patient’s quality of life remains to be determined.

## Conclusion

The present study provides evidence of perimetric defects in a group of PD patients without concomitant signs of anatomical retinal damage. Despite the absence of morphological evidence of RNFL tissue loss, the pattern of VF defects may resemble retinal ganglion cell dysfunction. Prospective longitudinal investigations would be valuable to elucidate retinal structural and functional changes in the course of the disease.

## Abbreviations

PD: Parkinson Disease; VF: Visual Field; OCT: Optical Coherence Tomography; RNFL: Retinal Nerve Fiber Layer; MD: Mean Deviation; PSD: Pattern Standard Deviation; IOP: Intraocular Pressure; MMSE: Mini Mental State Examination; VEP: Visual Evoked Potential; ERG: Electoretinogram; GHT: Glaucoma Hemifield Test; MSS: Mean Sensitivity Superior; MSI: Mean Sensitivity Inferior.

## Competing interests

The authors declare that they have no competing interests.

## Authors’ contribution

EET: Study design, revising the manuscript, interpretation of data, study coordination and supervision AID: Drafting and revising the manuscript, interpretation of data, statistical analysis AK: Drafting the manuscript, interpretation of dataED: Drafting the manuscript, analysis of dataFZ, SV, GP and SR: Acquisition and analysis of data GMH: Analysis and interpretation of data, revising the manuscript. All authors read and approved the final manuscript.

## Funding

There was no financial support for this study.

## Pre-publication history

The pre-publication history for this paper can be accessed here:

http://www.biomedcentral.com/1471-2415/12/54/prepub

## Supplementary Material

Additional file 1 Clinical characteristics of patients with Parkinson’s disease. Click here for file

## References

[B1] Chaudhuri KR Schapira AH Non-motor symptoms of Parkinson's disease: dopaminergic pathophysiology and treatment Lancet Neurol 2009 8 464 474 10.1016/S1474-4422(09)70068-7 19375664

[B2] Jones RD Donaldson IM Timmings PL Impairment of high-contrast visual acuity in Parkinson's disease Mov Disord 1992 7 232 238 10.1002/mds.870070308 1620141

[B3] Silva MF Faria P Regateiro FS Forjaz V Januário C Freire A Independent patterns of damage within magno-, parvo- and koniocellular pathways in Parkinson's disease Brain 2005 128 2260 2271 10.1093/brain/awh581 16000338

[B4] Bodis-Wollner I Marx MS Mitra S Bobak P Mylin L Yahr M Visual dysfunction in Parkinson's disease Loss in spatiotemporal contrast sensitivity Brain 1987 110 1675 1698 10.1093/brain/110.6.1675 3427405

[B5] Armstrong RA Visual symptoms in Parkinson's disease Parkinsons Dis 2011 2011 908306 21687773 10.4061/2011/908306 PMC3109513

[B6] Archibald NK Clarke MP Mosimann UP Burn DJ The retina in Parkinson's disease Brain 2009 132 1128 1145 10.1093/brain/awp068 19336464

[B7] Witkovsky P Dopamine and retinal function Doc Ophthalmol 2004 108 17 40 15104164 10.1023/b:doop.0000019487.88486.0a

[B8] Nguyen-Legros J Functional neuroarchitecture of the retina: hypothesis on the dysfunction of retinal dopaminergic circuitry in Parkinson's disease Surg Radiol Anat 1988 10 137 144 10.1007/BF02307822 3135618

[B9] Harnois C Di Paolo T Decreased dopamine in the retinas of patients with Parkinson's disease Invest Ophthalmol Vis Sci 1990 31 2473 2475 2243012

[B10] Inzelberg R Ramirez JA Nisipeanu P Ophir A Retinal nerve fiber layer thinning in Parkinson disease Vision Res 2004 44 2793 2797 10.1016/j.visres.2004.06.009 15342223

[B11] Altintas O Işeri P Ozkan B Cağlar Y Correlation between retinal morphological and functional findings and clinical severity in Parkinson's disease Doc Ophthalmol 2008 116 137 146 10.1007/s10633-007-9091-8 17962989

[B12] Moschos MM Tagaris G Markopoulos I Margetis I Tsapakis S Kanakis M Morphologic changes and functional retinal impairment in patients with Parkinson disease without visual loss Eur J Ophthalmol 2011 21 24 29 10.5301/EJO.2010.1318 20602324

[B13] Swanson EA Izatt JA Hee MR Huang D Lin CP Schuman JS In vivo retinal imaging by optical coherence tomography Opt Lett 1993 18 1864 1866 10.1364/OL.18.001864 19829430

[B14] Aaker GD Myung JS Ehrlich JR Mohammed M Henchcliffe C Kiss S Detection of retinal changes in Parkinson's disease with spectral-domain optical coherence tomography Clin Ophthalmol 2010 4 1427 1432 21188154 10.2147/OPTH.S15136 PMC3000768

[B15] Archibald NK Clarke MP Mosimann UP Burn DJ Retinal thickness in Parkinson's disease Parkinsonism Relat Disord 2011 17 431 436 10.1016/j.parkreldis.2011.03.004 21454118

[B16] Albrecht P Müller AK Südmeyer M Ferrea S Ringelstein M Cohn E Optical coherence tomography in parkinsonian syndromes PLoS One 2012 7 4 34891 Epub 2012 Apr 13 10.1371/journal.pone.0034891 PMC3325949 22514688

[B17] Yenice O Onal S Midi I Ozcan E Temel A I-Gunal D Visual field analysis in patients with Parkinson's disease Parkinsonism Relat Disord 2008 14 193 198 10.1016/j.parkreldis.2007.07.018 17888714

[B18] Gelb DJ Oliver E Gilman S Diagnostic criteria for Parkinson disease Arch Neurol 1999 56 33 39 10.1001/archneur.56.1.33 9923759

[B19] Goldberg I Graham SL Klistorner AI Multifocal objective perimetry in the detection of glaucomatous field loss Am J Ophthalmol 2002 133 29 39 10.1016/S0002-9394(01)01294-6 11755837

[B20] Murdoch IE Morris SS Cousens SN People and eyes: statistical approaches in ophthalmology Br J Ophthalmol 1998 82 971 973 10.1136/bjo.82.8.971 9828786 PMC1722711

[B21] Yavas GF Yilmaz O Küsbeci T Oztürk F The effect of levodopa and dopamine agonists on optic nerve head in Parkinson disease Eur J Ophthalmol 2007 17 812 816 17932860 10.1177/112067210701700520

[B22] Hajee ME March WF Lazzaro DR Wolintz AH Shrier EM Glazman S Inner retinal layer thinning in Parkinson disease Arch Ophthalmol 2009 127 737 741 10.1001/archophthalmol.2009.106 19506190

[B23] Huang D Swanson EA Lin CP Schuman JS Stinson WG Chang W Optical coherence tomography Science 1991 254 1178 1181 10.1126/science.1957169 1957169 PMC4638169

[B24] Sung KR Kim JS Wollstein G Folio L Kook MS Schuman JS Imaging of the retinal nerve fibre layer with spectral domain optical coherence tomography for glaucoma diagnosis Br J Ophthalmol 2011 95 909 914 10.1136/bjo.2010.186924 21030413 PMC3421150

[B25] Wojtkowski M Srinivasan V Fujimoto JG Ko T Schuman JS Kowalczyk A Three-dimensional retinal imaging with high-speed ultrahigh-resolution optical coherence tomography Ophthalmology 2005 112 1734 1746 10.1016/j.ophtha.2005.05.023 16140383 PMC1939719

[B26] Nassif N Cense B Park BH Yun SH Chen TC Bouma BE In vivo human retinal imaging by ultrahigh-speed spectral domain optical coherence tomography Opt Lett 2004 29 480 482 10.1364/OL.29.000480 15005199

[B27] Sehi M Grewal DS Sheets CW Greenfield DS Diagnostic ability of Fourier-domain vs time-domain optical coherence tomography for glaucoma detection Am J Ophthalmol 2009 148 597 605 10.1016/j.ajo.2009.05.030 19589493 PMC2784699

[B28] Chang RT Knight OJ Feuer WJ Budenz DL Sensitivity and specificity of time-domain versus spectral-domain optical coherence tomography in diagnosing early to moderate glaucoma Ophthalmology 2009 116 2294 2299 10.1016/j.ophtha.2009.06.012 19800694

[B29] Moreno-Montanes J Olmo N Alvarez A Garcia N Zarranz-Ventura J Cirrus high-definition optical coherence tomography compared with Stratus optical coherence tomography in glaucoma diagnosis Invest Ophthalmol Vis Sci 2010 51 335 343 10.1167/iovs.08-2988 19737881

[B30] Bayer AU Keller ON Ferrari F Maag KP Association of glaucoma with neurodegenerative diseases with apoptotic cell death: Alzheimer's disease and Parkinson's disease Am J Ophthalmol 2002 133 135 137 10.1016/S0002-9394(01)01196-5 11755850

[B31] Bodis-Wollner I Visual deficits related to dopamine deficiency in experimental animals and Parkinson's disease patients Trends Neurosci 1990 13 296 302 10.1016/0166-2236(90)90113-O 1695407

[B32] Djamgoz MB Hankins MW Hirano J Archer SN Neurobiology of retinal dopamine in relation to degenerative states of the tissue Vision Res 1997 37 3509 3529 10.1016/S0042-6989(97)00129-6 9425527

[B33] Peppe A Stanzione P Pierelli F De Angelis D Pierantozzi M Bernardi G Visual alterations in de novo Parkinson's disease: pattern electroretinogram latencies are more delayed and more reversible by levodopa than are visual evoked potentials Neurology 1995 45 1144 1148 10.1212/WNL.45.6.1144 7783879

[B34] Gottlob I Schneider E Heider W Skrandies W Alteration of visual evoked potentials and electroretinograms in Parkinson's disease Electroencephalogr Clin Neurophysiol 1987 66 349 357 10.1016/0013-4694(87)90032-0 2435514

[B35] Langheinrich T Elst L Tebartz V Lagrèze WA Bach M Lücking CH Visual contrast response functions in Parkinson's disease: evidence from electroretinograms, visually evoked potentials and psychophysics Clin Neurophysiol 2000 111 66 74 10.1016/S1388-2457(99)00223-0 10656512

[B36] Nightingale S Mitchell KW Howe JW Visual evoked cortical potentials and pattern electroretinograms in Parkinson's disease and control subjects J Neurol Neurosurg Psychiatry 1986 49 1280 1287 10.1136/jnnp.49.11.1280 3794734 PMC1029077

[B37] Bodis-Wollner I Yahr MD Measurements of visual evoked potentials in Parkinson's disease Brain 1978 101 661 671 10.1093/brain/101.4.661 737524

[B38] Shibasaki H Tsuji S Kuroiwa Y Oculomotor abnormalities in Parkinson's disease Arch Neurol 1979 36 360 364 10.1001/archneur.1979.00500420070009 454234

[B39] Wild JM Pacey IE Hancock SA Cunliffe IA Between-algorithm, between-individual differences in normal perimetric sensitivity: full threshold, FASTPAC, and SITA. Swedish Interactive Threshold algorithm Invest Ophthalmol Vis Sci 1999 40 1152 1161 10235548

[B40] Bengtsson B Olsson J Heijl A Rootzén H A new generation of algorithms for computerized threshold perimetry, SITA Acta Ophthalmol Scand 1997 75 368 375 9374242 10.1111/j.1600-0420.1997.tb00392.x

[B41] Dalrymple-Alford JC MacAskill MR Nakas CT Livingston L Graham C Crucian GP The MoCA: well-suited screen for cognitive impairment in Parkinson disease Neurology 2010 9 75 1717 1725 21060094 10.1212/WNL.0b013e3181fc29c9

