# Supplementary material for: Perimetric and retinal nerve fiber layer findings in patients with Parkinson’s disease
Source: BMC Ophthalmol. 2012 Oct 2;12:54. doi: 10.1186/1471-2415-12-54 (PMC3515471; doi:10.1186/1471-2415-12-54)
Supplement: Additional file 1 — Clinical characteristics of patients with Parkinson’s disease. [file 1471-2415-12-54-S1.doc]

**Additional File 1**

|  | **Sex** | **Age at**  **Examination (years)** | **Initial symptoms** | **Duration of PD (years)** | **Modified Hoehn and Yahr (H-Y) stage** | **Mini-Mental State examination** |
| --- | --- | --- | --- | --- | --- | --- |
|
| Patient 1 | Man | 66 | Rigidity in the right upper limb | 6 | 2.5 | 30 |
| Patient 2 | Woman | 72 | Resting tremor in the upper limbs | 4 | 2 | 27 |
| Patient 3 | Man | 62 | Left-sided rigidity and bradykinesia | 5 | 2 | 30 |
| Patient 4 | Woman | 60 | Right-sided resting tremor | 3 | 1 | 30 |
| Patient 5 | Man | 73 | Resting tremor in the right upper limb | 6 | 1.5 | 25 |
| Patient 6 | Man | 37 | Resting tremor in the left upper limb | 2 | 1 | 30 |
| Patient 7 | Woman | 73 | Left-sided rigidity | 17 | 2 | 28 |
| Patient 8 | Man | 71 | Right-sided resting tremor, rigidity | 3 | 2.5 | 30 |
| Patient 9 | Woman | 69 | Right-sided rigidity, bradykinesia | 4 | 2 | 29 |
| Patient 10 | Woman | 61 | Resting tremor in the left upper limb | 4 | 2 | 30 |
| Patient 11 | Man | 73 | Resting tremor in the right upper limb | 9 | 2 | 30 |
| Patient 12 | Man | 57 | Right-sided tremor, rigidity, bradykinesia | 5 | 2 | 30 |
| Patient 13 | Woman | 69 | Resting tremor in left upper limb | 5 | 2.5 | 30 |
| Patient 14 | Woman | 70 | Right-sided rigidity, bradykinesia | 1 | 1 | 29 |
| Patient 15 | Man | 71 | Left-sided tremor, rigidity | 6 | 2.5 | 30 |
| Patient 16 | Woman | 84 | Right-sided tremor, rigidity, bradykinesia | 8 | 2.5 | 28 |
| Patient 17 | Man | 50 | Left-sided tremor, rigidity | 2 | 1 | 28 |
| Patient 18 | Man | 77 | Left-sided tremor, rigidity | 6 | 2 | 30 |
| Patient 19 | Man | 73 | Resting tremor in the right upper limb | 10 | 3 | 30 |
| Patient 20 | Woman | 72 | Resting tremor in the right upper limb | 7 | 2.5 | 22 |
| Patient 21 | Man | 73 | Left-sided tremor, rigidity, bradykinesia | 8 | 2.5 | 24 |
| Patient 22 | Man | 67 | Resting tremor in the upper limbs | 3 | 2 | 27 |
| Patient 23 | Woman | 64 | Right-sided resting tremor | 2 | 1 | 29 |
| Patient 24 | Man | 52 | Resting tremor in right upper limb | 1 | 1 | 24 |
